# Associations of left atrial volumes and Doppler filling indices with left atrial function in acute myocardial infarction

**DOI:** 10.1111/cpf.12533

**Published:** 2018-07-01

**Authors:** Egil Henriksen, Jonas Selmeryd, Pär Hedberg

**Affiliations:** ^1^ Department of Clinical Physiology Västmanland County Hospital Västerås Sweden; ^2^ Centre for Clinical Research Uppsala University Västmanland County Hospital Västerås Sweden

**Keywords:** diastolic function, Doppler indices, echocardiography, left atrium, myocardial infarction

## Abstract

Recent findings suggest that left atrial (LA) function is more strongly related to adverse prognosis than LA volumes. We aimed to evaluate the associations between LA volumes and Doppler filling indices with LA function. Echocardiographic LA volumes (LAVs), mitral valve early (MV‐E) and late (MV‐A) peak flow velocities, and mitral atrioventricular plane tissue‐Doppler early (TD‐e′) and late (TD‐a′) peak velocities were obtained in 320 patients with acute myocardial infarction (AMI) free from atrial fibrillation and more than moderate valvular disease. LA function was estimated as the LA emptying fraction (LAEF), that is 100× (LAVmax‐LAVmin)/LAVmax. LA reservoir volume was calculated as LAVmax‐LAVmin and LA transit volume as LV stroke volume‐reservoir volume. In restricted cubic spline regression analyses with multivariable adjustment, a reduced LAEF was strongly associated with smaller reservoir volume, larger transit volume, LAVmax, LAVpreA and especially LAVmin. MV‐E linearly increased with a lower LAEF, whereas MV‐A decreased but only below LAEF levels of approximately 45%. The resulting E/A ratio showed a sudden increase in LAEF levels below ~45%. Lower TD‐a′ was linearly associated with a lower LAEF. In conclusion, a reduced atrial function was associated with smaller LA reservoir volume, larger LA transit volume, lower TD‐a′, a non‐linear decrease in MV‐A and a non‐linear increase in E/A. Our findings are likely a reflection of the adaptation to sustain LV filling volume and counteracting a rise in pulmonary venous pressure in face of an enhanced LV end‐diastolic pressure.

## Introduction

The left atrium (LA) provides several functions that influence the overall cardiac performance. During left ventricular (LV) systole, the LA serves as a reservoir storing pulmonary venous blood that passively empties into the LV after the mitral valve opening. Throughout the diastasis, the LA acts as a transit chamber permitting passive flow from the pulmonary veins into the LV. Finally, the LA is a contractile chamber that actively contributes to the final LV end‐diastolic pressure (LVEDP) and volume. The reservoir, passive emptying, transit and LA contraction phases permit a more or less continuous venous flow through the pulmonary circulation regardless of the intermittent LV filling (Stefanadis *et al*., 2001).

There are growing evidence suggesting that LA structural and functional changes play an important role in both risk stratification and symptom progression in various patients groups (Hoit, 2014). LA maximum volume indexed for body surface area (LAVImax) is a well‐recognized prognostic marker in different patient groups and community‐based populations (Benjamin *et al*., 1995; Tsang *et al*., 2003; Gupta *et al*., 2013). Recent findings suggest that LA minimum volume index (LAVImin) is more strongly related to LV filling disorders and is a better prognostic marker of future cardiovascular events than LAVImax (Appleton *et al*., 1993; Fatema *et al*., 2009; Caselli *et al*., 2010; Russo *et al*., 2012; Hedberg *et al*., 2016, 2017). We have shown that the LA emptying fraction (LAEF), measured as (LVImax‐LAVImin)/LAVImax, is even more strongly related to cardiovascular events than its component LA phasic volumes in a community‐based cohort (Hedberg *et al*., 2017). The reason for these findings is unknown but suggests that a reduced LAEF is a more robust marker of LV filling disorders than single LA volume measures.

We aimed to explore the associations of LA phasic volumes and Doppler filling indices with LA function measured as LAEF in patients with acute myocardial infarction (AMI).

## Method

### Study population

The participants were recruited from the Västmanland Myocardial Infarction Study (VaMIS; ClinicalTrials.gov number, NCT01452178). Details of the design and study population have been reported elsewhere (Eriksson Östman *et al*., 2017). In short, consecutive patients hospitalized for AMI from November 2005 to May 2011 were included in the VaMIS study. All subjects underwent clinical examination, electrocardiography (ECG), echocardiographic examination and blood sampling.

In the VaMIS cohort (*n* = 1008), all LA volume measures were obtained consecutively in 691 patients. Subjects with atrial fibrillation (*n* = 75) or more than moderate valvular disease (*n* = 11) were excluded. Further, patients with missing values on any of the analysed variables (mainly because of reduced echocardiographic image quality) were excluded (*n* = 285). Finally, 320 subjects (232 men and 88 women) were included in the analyses. A written informed consent was obtained from all participants. The ethical committee of Uppsala, Sweden, approved the study (Protocol number 2005:169).

### Echocardiographic image acquisition

A two‐dimensional (2D) echocardiographic examination was performed by experienced echocardiographers using a commercially available system (Vingmed Vivid Seven, General Electric, Horten, Norway). The images were obtained in the left lateral recumbent position using a phased array transducer in the standard parasternal and apical views. The ECG‐triggered 2D images and Doppler data were stored digitally in a cine loop format. Three consecutive cardiac cycles were recorded during quiet breathing.

### Echocardiographic measures

All measurements were performed by an experienced echocardiographer (PH). LV dimensions were measured from the parasternal long‐axis view, and LV mass was calculated using the ASE‐recommended formula (Lang *et al*., 2015). LV end‐diastolic and end‐systolic volumes were measured from the apical four‐ and two‐chamber views, and LV stroke volume and LV ejection fraction (LVEF) were calculated according to the biplane Simpson's rule (Lang *et al*., 2015). In the assessment of LA volumes, the single‐plane disk method was used in the apical four‐chamber view. The stored loops of this view were dedicated to LA visualization and oriented to maximize the LA area. LAVmax (i.e., end systolic) assessment was performed using the frame immediately preceding the mitral valve opening and LAVmin (i.e., end diastolic) was obtained using the frame contiguous to mitral valve closure. The LA volume measured prior to the beginning of the p wave on the ECG was recorded as LAVpreA. The LA function was expressed as LAEF and calculated as 100 × (LAVmax − LAVmin)/LAVmax. The LA passive emptying volume was measured as LAVmax − LAVpreA. The LA contraction volume was defined as LAVpreA − LAVmin. The LA reservoir volume was estimated as LAVmax − LAVmin and the LA transit volume as LV stroke volume − LA reservoir volume. The total early filling volume was calculated as LAVmax − LAVpreA + LA transit volume. All measured and calculated LA volumes were indexed for body surface area.

Mitral inflow was recorded using pulsed Doppler at the tips of the mitral leaflets. The peak early (MV‐E) and late (MV‐A) transmitral diastolic flow velocities, the E/A ratio and the deceleration time of the early filling velocity (MV‐Edt) were obtained. The peak velocities of the early diastolic wave (TD‐e′) and the atrial contraction wave (TD‐a′) were measured using pulsed‐wave tissue Doppler with the sample volume close to the mitral valve annulus in the apical four‐chamber view in the septal and lateral walls and were averaged. The E/e′ ratio was calculated as MV‐E/TD‐e′.

### Biochemistry

After participants fasted overnight, venous blood samples were taken and immediately sent to the accredited Laboratory of Clinical Chemistry, Västmanland County Hospital, Västerås, Sweden. Plasma levels of NT‐proBNP were measured by a commercially available sandwich immunoassay using monoclonal antibodies and separation based on biotin‐streptavidin binding (Elecys 1010 and Cobas e411; Roche Diagnostics, Mannheim, Germany). The within‐run coefficients of variation were 3·1% and 3·6% for low and high levels of NT‐proBNP, respectively.

### Statistics

Data are presented as mean ± standard deviation for continuous variables and as numbers and percentages for categorical variables. The participants were categorized in quartiles according to LAEF. Continuous and categorical variables were compared across quartiles using analysis of variance and the Fisher exact test, respectively. NT‐proBNP showed a highly skewed distribution and was compared across quartiles of LAEF with the Kruskal–Wallis test. Univariate associations between LAEF and various variables were assessed using Pearson's correlation coefficients. Differences between dependent and overlapping correlation coefficients were tested according to Steiger using the ‘cocor’ package for R (Steiger, 1980). Multiple linear regression models were used to evaluate the associations of LAEF with various LA phasic volumes or Doppler indices with multivariable adjustment for age, sex, smoking, body mass index, diastolic blood pressure, diabetes, previous myocardial infarction, previous TIA/stroke, LVEF, LV mass index, moderate (versus less than moderate) valvular disease and NT‐proBNP. Logarithmic transformation was applied to NT‐proBNP because of a skewed distribution. The potential nonlinearity in these associations was addressed by restricted cubic splines with three knots located at the 10th, 50th and 90th percentiles corresponding to LAEF values of 26%, 46% and 59%, respectively. A Wald test was used for the regression coefficients of LAEF (i.e. linear and spline terms) to test the null hypothesis that there was no association between LAEF and the dependent variable in focus. The same test was used for the coefficients of the spline terms only to test the null hypothesis that the association was linear.

Stata version 15 (StataCorp LP, College Station, TX, USA) and R version 3·4·3 (R Foundation for Statistical Computing, Vienna, Austria) were used for the analyses. A *P* value <0·05 was considered significant.

## Results

### Patient characteristics

The basic characteristics of the study population according to quartiles of LAEF are given in Table 1. The study sample included 320 patients with AMI of whom 232 were men and 88 women with a mean age of 67·4 ± 11·6 and 73·3 ± 11·5 years, respectively. The excluded patients had missing values on mainly Simpson‐LVEF (*n* = 165, 27·3%) and LA volumes (*n* = 74, 12·2%). Compared with the included patients, the excluded patients were more often women (38·4% versus 27·8%, *P *=* *0·006) and had a larger body mass index (27·5 versus 26·6 kg m^−2^, *P *=* *0·015). There were no significant differences between the included and excluded patients regarding age, smoking, hypertension, diabetes, previous myocardial infarction or previous TIA/stroke.

**Table 1:** Basic characteristics according to quartiles (Q4–Q1) of left atrial emptying fraction (LAEF) in 320 patients with acute myocardial infarction

	Quartiles of LAEF				*P* value
	Q4 >53% *n* = 80	Q3 46%–53% *n* = 80	Q2 35%–45% *n* = 80	Q1 <35% *n* = 80	
Male sex, *n* (%)	66 (82·5)	61 (76·3)	55 (68·8)	50 (62·5)	0·027
Age (years)	63·5 ± 10·4	67·6 ± 11·0	70·5 ± 11·9	74·5 ± 11·3	<0·001
Smoker, *n* (%)	19 (23·8)	19 (23·8)	16 (20·0)	14 (17·5)	0·715
Body mass index (kg m^−2^)	26·8 ± 4·1	26·3 ± 3·8	27·0 ± 4·5	26·2 ± 4·0	0·537
Systolic BP (mmHg)	125 ± 17	131 ± 19	127 ± 22	126 ± 23	0·279
Diastolic BP (mmHg)	72·5 ± 11·1	76·0 ± 9·6	69·9 ± 11·2	69·9 ± 11·4	<0·001
Pulse pressure (mmHg)	52·6 ± 11·0	54·8 ± 14·8	56·6 ± 18·3	56·1 ± 17·2	0·365
Heart rate (bpm)	62·2 ± 9·9	64·1 ± 11·3	62·9 ± 10·3	65·5 ± 12·0	0·246
Treatment for hypertension, *n* (%)	29 (36·3)	40 (50·0)	40 (50·0)	49 (61·3)	0·018
Diabetes, *n* (%)	9 (11·3)	14 (17·5)	15 (18·8)	17 (21·3)	0·382
Previous MI, *n* (%)	12 (15·0)	19 (23·8)	21 (26·3)	18 (22·5)	0·339
Previous TIA/stroke, *n* (%)	3 (3·8)	0 (0·0)	8 (10·0)	11 (13·8)	<0·001
NT‐proBNP (ng l^−1^)^a^	622 (218, 1253)	1079 (376, 2174)	1189 (544, 2960)	2464 (1242, 7232)	<0·001

BP, blood pressure; LAEF, left atrial emptying fraction; MI, myocardial infarction; NT‐proBNP, N‐terminal pro‐B‐type natriuretic peptide; TIA, transitory ischemic attack.

a Values are median (25th percentile, 75th percentile).

### LV volume indices according to quartiles of LAEF

The LV end‐diastolic and end‐systolic volume indices were significantly larger and LVEF significantly lower in the lowest compared with the highest quartile of LAEF (Table 2). However, LV stroke volume did not differ between quartiles of LAEF.

**Table 2:** Echocardiographic characteristics according to quartiles (Q4–Q1) of left atrial emptying fraction (LAEF) in 320 patients with acute myocardial infarction

	Quartiles of LAEF				*P* value
	Q4 >53% *n* = 80	Q3 46%–53% *n* = 80	Q2 35%–45% *n* = 80	Q1 <35% *n* = 80	
LV ejection fraction (%)	59·6 ± 9·3	57·0 ± 10·4	54·8 ± 12·6	49·1 ± 15·0	<0·001
LV mass index (g m^−2^)	115 ± 29	116 ± 30	123 ± 30	144 ± 42	<0·001
Moderate valvular disease, *n* (%)	0 (0·0)	3 (3·8)	10 (12·5)	10 (12·5)	<0·001
MV filling time (ms)	563 ± 156	522 ± 138	548 ± 146	516 ± 164	0·177
MV‐E (cm s^−^)	58·4 ± 14·0	62·3 ± 16·9	65·5 ± 18·5	69·9 ± 24·7	0·001
MV‐A (cm s^−1^)	62·6 ± 16·3	72·7 ± 22·8	66·0 ± 22·8	65·5 ± 25·8	0·032
E/A ratio	0·98 ± 0·28	0·91 ± 0·33	1·09 ± 0·44	1·26 ± 0·80	<0·001
MV‐E DT (ms)	225 ± 66	216 ± 61	218 ± 78	216 ± 87	0·860
TD‐e′ (cm s^−1^)	7·64 ± 2·05	6·78 ± 1·82	6·39 ± 2·06	5·94 ± 1·86	<0·001
TD‐a′ (cm s^−1^)	8·74 ± 1·74	8·40 ± 1·67	7·20 ± 1·94	6·21 ± 2·32	<0·001
E/e′ ratio	7·96 ± 2·14	9·55 ± 2·83	11·2 ± 4·8	12·6 ± 5·2	<0·001
LVEDV index (ml m^−2^)	45·4 ± 11·1	46·2 ± 17·6	47·6 ± 12·5	55·8 ± 25·1	<0·001
LVESV index (ml m^−2^)	18·8 ± 7·8	20·9 ± 15·6	22·4 ± 10·9	31·2 ± 24·9	<0·001
Stroke volume index (ml m^−2^)	26·6 ± 6·0	25·3 ± 5·4	25·2 ± 5·9	24·5 ± 5·9	0·140
LAVI max (ml m^−2^)	30·9 ± 8·7	30·3 ± 7·4	36·7 ± 11·7	39·3 ± 12·8	<0·001
LAVI pre‐A (ml m^−2^)	21·4 ± 6·4	23·3 ± 6·6	29·6 ± 9·4	35·2 ± 11·9	<0·001
LAVI min (ml m^−2^)	12·7 ± 3·6	15·3 ± 3·7	21·7 ± 7·2	29·5 ± 11·4	<0·001
LAEF (%)	58·8 ± 4·2	49·5 ± 2·2	41·1 ± 3·1	25·8 ± 8·2	<0·001
LAVI reservoir (ml m^−2^)	18·2 ± 5·6	15·0 ± 3·8	15·0 ± 4·7	9·80 ± 4·11	<0·001
Transit volume index (ml m^−2^)	8·41 ± 6·32	10·3 ± 5·7	10·1 ± 7·3	14·7 ± 6·6	<0·001
LA passive emptying index (ml m^−2^)	9·51 ± 4·71	7·00 ± 3·87	7·16 ± 4·63	4·10 ± 5·07	<0·001
LA total early filling index (ml m^−2^)	17·9 ± 6·4	17·3 ± 6·8	17·3 ± 6·8	18·8 ± 6·9	0·424
LAVI contraction (ml m^−2^)	8·69 ± 3·62	8·03 ± 4·04	7·86 ± 3·71	5·70 ± 4·72	<0·001

DT, deceleration time; LA, left atrium; LAVI, left atrial volume indexed for body surface area; LV, left ventricular; LVEDV, left ventricular end diastolic volume; LVESV, left ventricular end systolic volume; MV, mitral valve; TD, tissue Doppler.

### Left atrial phasic volume indices in association with LAEF

**Figure 1:**
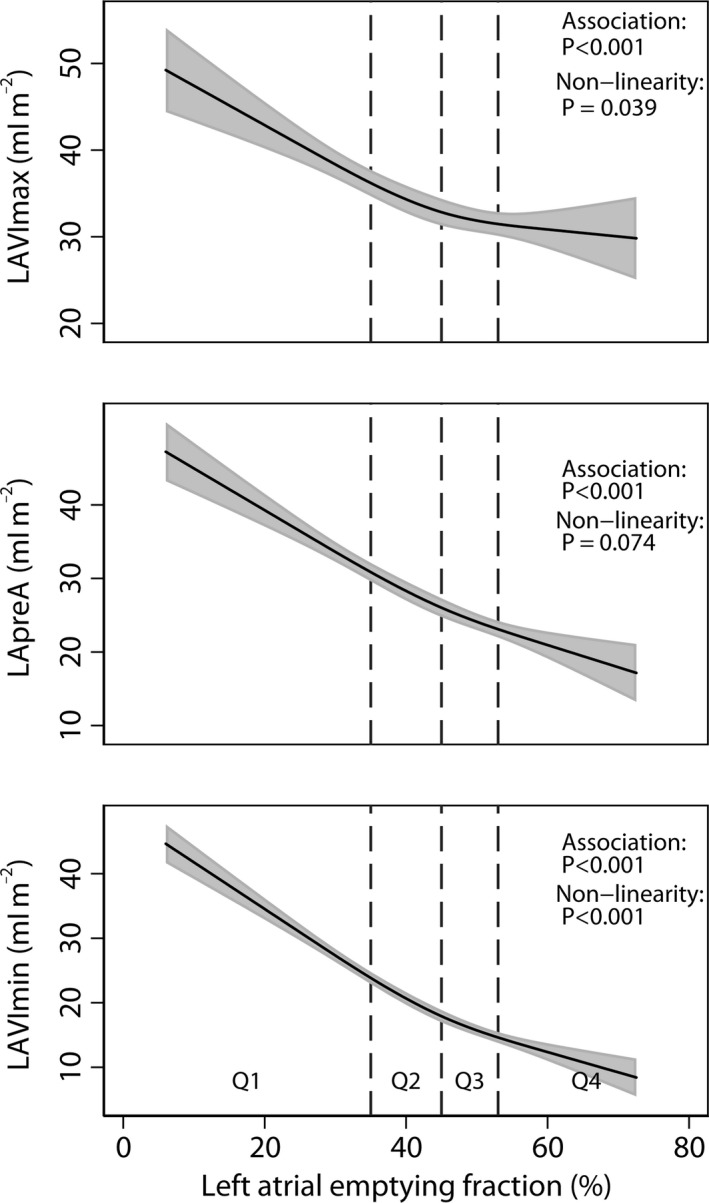
Associations of left atrial emptying fraction (LAEF) with maximum (LAVImax), pre‐A (LAVIpreA) and minimum (LAVImin) left atrial volumes indexed for body surface area, after multivariable adjustment for age, sex, smoking, body mass index, diastolic blood pressure, diabetes, previous myocardial infarction, previous TIA/stroke, LVEF, LV mass index, moderate valvular disease and NT‐proBNP. Results were obtained by multiple linear regression with restricted cubic splines with three knots for LAEF. *P* values are for Wald tests of LAEF linear and spline terms jointly (association) and LAEF spline terms only (non‐linearity). Vertical dashed lines indicate borders between quartiles (Q1‐Q4) of LAEF.

LAVImax, LAVIpreA and LAVImin were on average 8 ml m^−2^ (27%), 14 ml m^−2^ (64%) and 17 ml m^−2^ (132%) larger, respectively, in the lowest compared with the highest quartile of LAEF (Table 2). The upper panel of Fig. 1 shows the association between LAEF and LAVImax after multivariable adjustment for age, sex, smoking, body mass index, diastolic blood pressure, diabetes, previous myocardial infarction, previous TIA/stroke, LVEF, LV mass index, moderate valvular disease and NT‐proBNP. The association was significant (*P*<0·001), and there was evidence of a non‐linear relation (*P *=* *0·039). The corresponding associations of LAEF with LAVIpreA and LAVImin (middle and lower panels of Fig. 1) were highly significant (both *P*<0·001), and there was evidence for a non‐linear relationship of LAEF with LAVImin (*P*<0·001) but not with LAVIpreA (*P *=* *0·074). LAVImax and LAVImin were highly correlated (*r *=* *0·87), but the correlation between LAEF and LAVImin was significantly higher than between LAEF and LAVImax (*r *=* *−0·75 versus *r *=* *−0·37; *P*<0·001).

**Figure 2:**
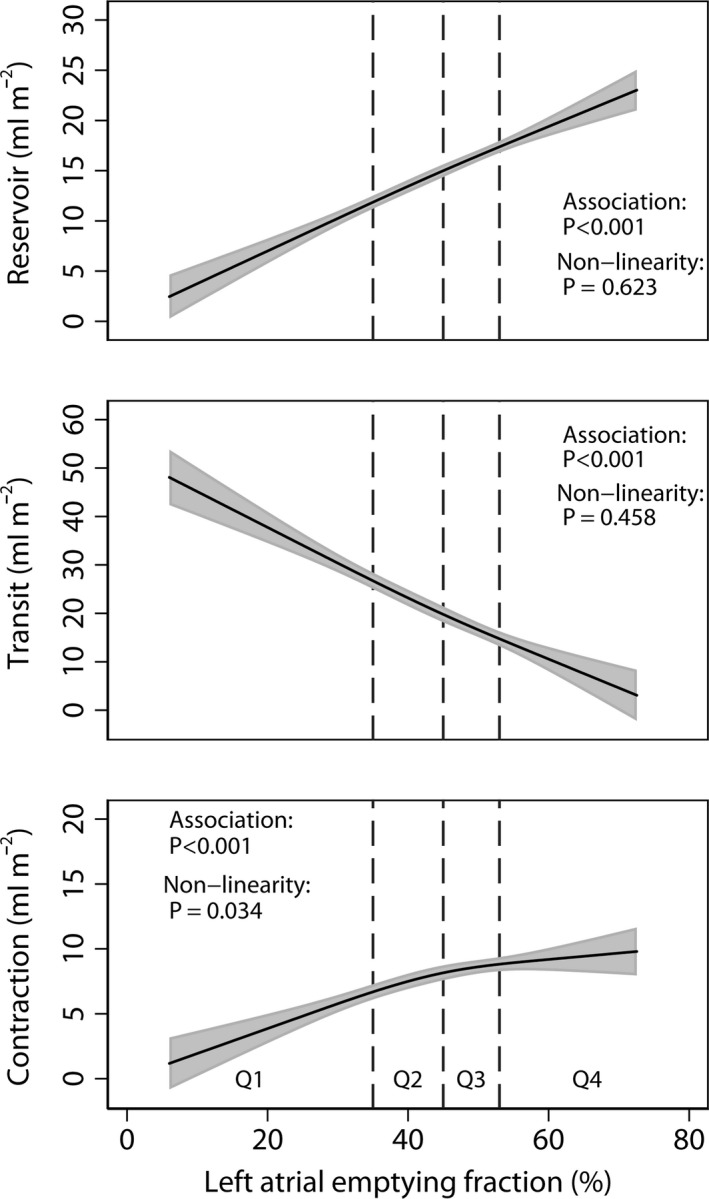
Associations of left atrial emptying fraction (LAEF) with left atrial reservoir, transit and contraction volumes indexed for body surface area after multivariable adjustment. For further details see Fig. 1.

The LA reservoir volume index was on average 8 ml m^−2^ (46%) lower, the LA transit volume index 6 ml m^−2^ (75%) higher and the LA contraction volume index 3 ml m^−2^ (34%) lower in the lowest compared with the highest quartile of LAEF (Table 2). After multivariable adjustment, these indices were highly significantly associated with LAEF (all *P*<0·001; Fig. 2) and only the contraction volume index showed evidence of non‐linearity (*P *=* *0·034).

### Doppler filling indices in association with LAEF

**Figure 3:**
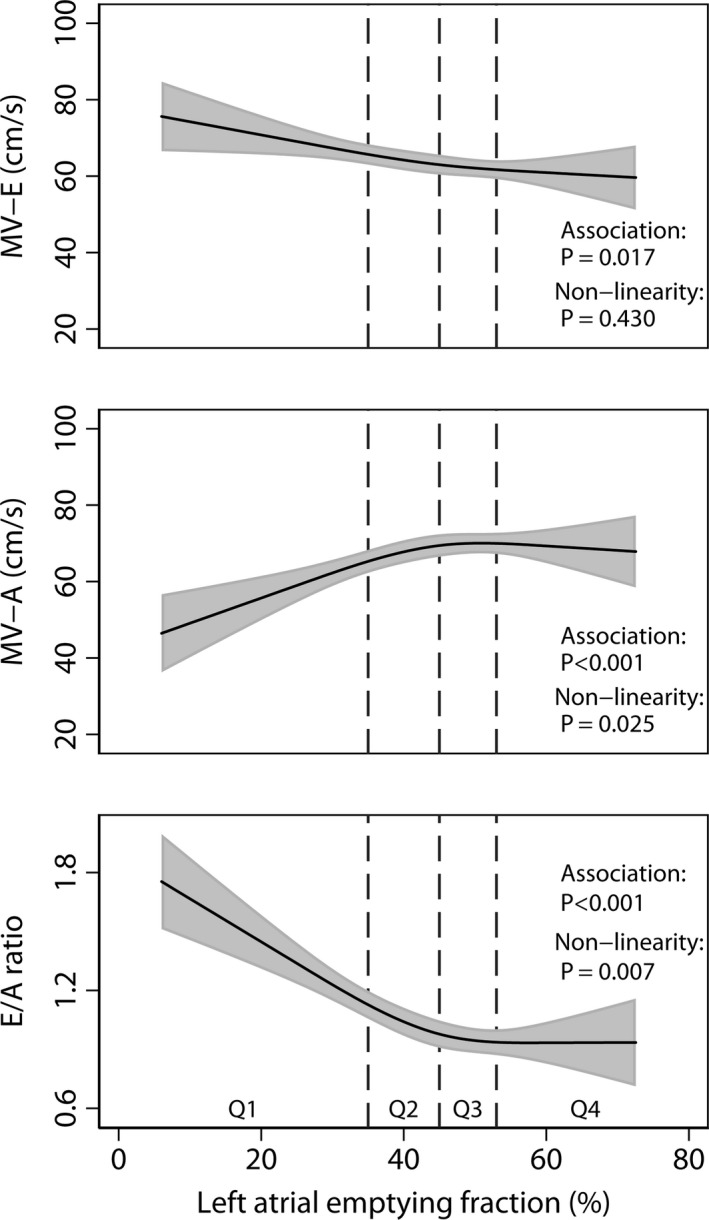
Associations of left atrial emptying fraction (LAEF) with peak early (MV‐E), peak late (MV‐A) transmitral flow velocities and the E/A ratio after multivariable adjustment. For further details see Fig. 1.

The findings of Doppler filling indices according to quartiles of LAEF are given in Table 2. After multivariable adjustment, the associations of MV‐E (*P *=* *0·017), MV‐A (*P*<0·001) and the E/A ratio (*P*<0·001) with LAEF were significant (Fig. 3) and these relationships were non‐linear for MV‐A (*P *=* *0·025) and E/A (*P *=* *0·007) but not for MV‐E (*P *=* *0·430).

**Figure 4:**
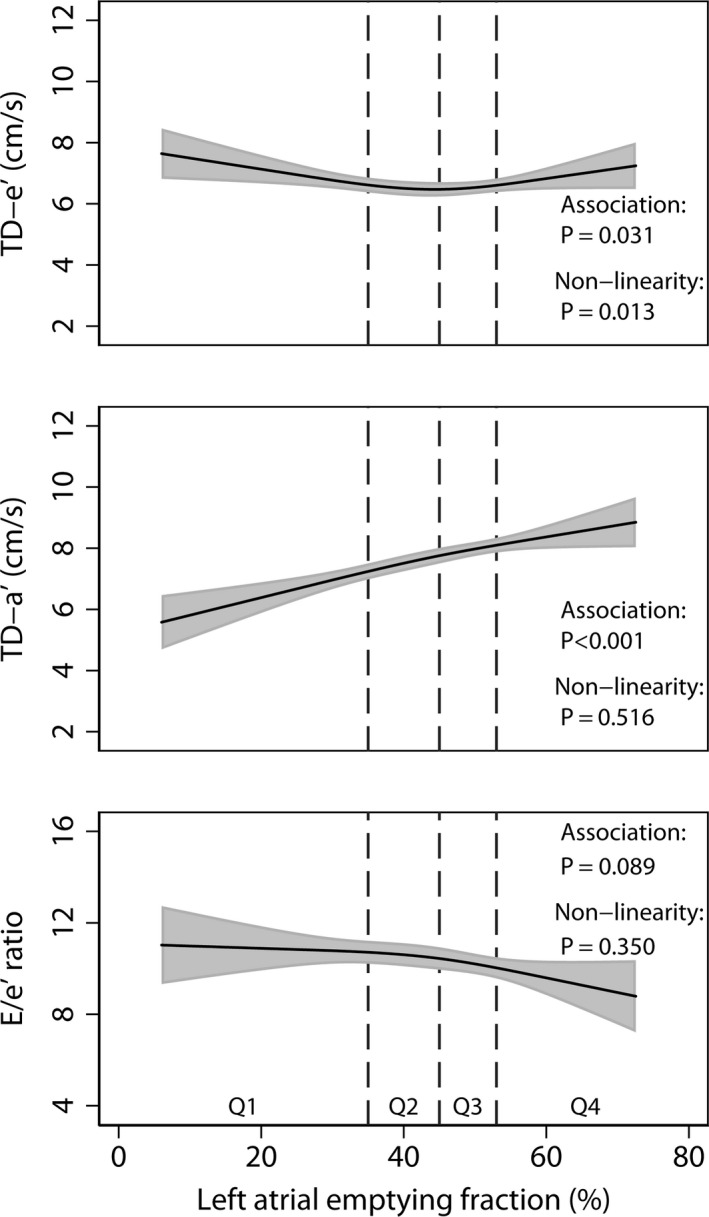
Associations of left atrial emptying fraction (LAEF) with peak early (TD‐e′), peak late (TD‐a′) mitral tissue Doppler velocities and the E/e′ ratio after multivariable adjustment. For further details see Fig. 1.

TD‐e′ was significantly related to LAEF (*P *=* *0·031) with evidence of a non‐linear association after multivariable adjustment (*P *=* *0·013; Fig. 4, upper panel). A lower TD‐a’ was strongly associated with a lower LAEF (*P*<0·001) without evidence of non‐linearity (Fig. 4, middle panel). E/e′ showed no significant association with LAEF after multivariable adjustment (*P *=* *0·089).

### Reproducibility

The reproducibility of the echocardiographic LV dimensions, LA volumes and Doppler measures in our laboratory has been published previously (Henriksen *et al*., 2015; Hedberg *et al*., 2016).

## Discussion

Attributable to a reduced operative LV compliance, it is a physiological paradigm that the increase in LVEDP is to optimize the myocardial wall‐tension/‐force relations to maintain the physiological demand of an adequate cardiac output (Braunwald & Frahm, 1961). The LVEDP must be kept high enough to reach the length dependency for an optimal contractile performance but low enough to prevent a significant augment in backward pressures. However, an elevated LVEDP can only be generated by an augment in the LA pressures and the thin‐walled LA cannot resist a prolonged pressure or volume load without compensatory changes in LA phasic volumes.

In the present study, a reduced LAEF was associated with a decrease in the LA reservoir and contraction volumes, and an increase in LA maximum, pre‐A, minimum and transit volumes. LAVImin was more strongly related to LAEF than LAVImax suggesting that a decline in LAEF is mainly driven by an augment in LAVImin. The restricted cubic spline analyses revealed that MV‐E linearly increased with a lower LAEF, whereas the MV‐A decreased but only below LAEF levels of approximately 45%. The resulting E/A ratio showed a sudden increase in LAEF levels below ~45%. A lower TD‐a′ was linearly associated with a lower LAEF, whereas E/e′ showed no significant association with LAEF after multivariable adjustment.

### Reservoir function

As both the LA reservoir volume and the LA V wave pressure (i.e. the LA pressure during ventricular systole) originates from ventricular systole, the separation of the cardiac cycle into the dichotomy of systole and diastole is arbitrary. In an open pericardial pig model, Barbier *et al*. (1999) showed that LA reservoir function was determined by the atrial relaxation, the descent of the atrioventricular plane and the LA stiffness. A relation between LA compliance and LA reservoir function has been demonstrated after LA appendectomy or appendage clamping and a direct relation between LA reservoir function and cardiac performance has been suggested (Suga, 1974; Hoit *et al*., 1993; Tabata *et al*., 1998). Despite a significant 46% lower reservoir volume index, our data showed that there was no significant difference in resting stroke volume index between the highest and lowest quartiles of LAEF. This finding can be explained by a marked compensatory increase in LA transit volume during early filling (Fig. 2) as previously demonstrated in invasive models (Hoit *et al*., 1993).

During even mild‐to‐moderate exercise, there is a marked decrease in diastolic filling time, merging the LV early and late filling which might affect the transit volume and thus the cardiac output reserve (Sundstedt *et al*., 2007). A low reservoir volume is a common finding in patients with heart failure and might contribute to the abnormal cardiac output response during even mild‐to‐moderate exercise observed in heart failure patients (Tan *et al*., 2009). Moreover, a low reservoir volume is linked to a reduced operative LA compliance generating an augment in peak LA V wave pressure and thereby an increase in systolic pulmonary artery pressure (Dernellis *et al*., 1998). When the reservoir volume is small, the pulmonary venous flow during ventricular systole fades or terminates which might fuel LA systolic blood stasis and contribute to an increased thrombogenicity even in the absence of atrial fibrillation. Interestingly, previous stroke was significantly more common in our patients in sinus rhythm with an LAEF below the median (LAEF <46%) than in those above the median (11·9% versus 1·9%; *P *=* *0·001).

In our AMI patients, the association of LAVImin with LAEF was significantly stronger than LAVImax. Dernellis *et al*. (1998) showed that LAVImax was smaller in AMI patients compared with congestive heart failure patients and that the LA pressure–volume relations were dissimilar between these patient groups. In the AMI patients, the LA V wave pressure was lower than the LA A wave pressure, whereas in congestive heart failure, the LA V wave pressure was high in relation to the LA A wave pressure. These findings suggest that the LA V wave pressure and disease duration is important determinants of LAVImax and that LAVImax possibly should be used with caution as an index of LV filling in AMI patients.

### Left atrial passive emptying and transit function

Accompanied by elastic energy stored in the LV during contraction, the energy stored in the LA during peak systole is released during early LV filling and the sum these forces might be important in propelling the AV‐plane towards the base of the heart during diastole and thus being key determinants of the LA passive emptying. A decline in LA passive emptying volume increases the LA pre‐A volume and subsequently raising the LA preload prior to LA contraction. Our data showed a significant but small rise in the MV‐E wave velocity with reduced LAEF suggesting an augment in the LA V wave pressure presumably initiated by a decreased operative LA compliance. Despite a 43% decline in the LA passive emptying volume index, the total early filling volume index remained unchanged. To preserve the total early filling volume index, the estimated LA transit volume index increased by 75% making early LV filling exceedingly dependent on the pulmonary venous transit volume.

### Active emptying function

In 26 patients with LV disease, Braunwald *et al*. observed a normal mean left atrial pressure (MLAP) despite a marked elevated LVEDP (Braunwald & Frahm, 1961). In a retrospective study of all patients undergoing right‐ and left‐heart catheterization at a large academic centre it was found that, among 5454 patients with a normal MLAP and a pulmonary capillary wedge pressure of <15 mmHg, 41% had an increased LVEDP of >15 mmHg (Halpern & Taichman, 2009). The likely explanation for the abovementioned observations is that the examined subjects had a preserved LA contractile function enabling a substantial increase in LA pressure during LA contraction (high peak LA A wave pressure), thereby avoiding a significant increase in MLAP. The present data showed that a low LAEF was associated with a high LA pre‐A volume. An augment in LA volume prior to the LA contraction can optimize the LA wall‐tension/‐force relations and so facilitate the LA contraction force. This might explain the modest decline in LA contraction volume regardless of a reduced LAEF. However, as the myocyte fibre shortening is limited there is a concomitant augment in LAVImin.

In our patients, the TD‐a′ peak velocity showed a positive and linear association with LAEF. In contrast, the MV‐A peak velocity seemed to decrease in parallel with LAEF only at lower LAEF levels. Our data suggest that the MV‐A peak velocity is a late marker of a reduced LA function. However, the TD‐a′ peak velocity was strongly and linearly related to LAEF, suggesting that it might be a more reliable marker of LA contractile function than the MV‐A. The observed decrease in MV‐A, TD‐a′ and LA contraction volume index can be explained by a deterioration of the LA contractile power and/or an augment in LV pressure during atrial contraction. Nevertheless, when the LA no longer can produce a high peak LA A wave pressure, the physiological reply is an upward shift in the LA pressure/volume relation producing an augment in the LA V wave pressure and MLAP.

So far, LAVImax is the guideline‐recommended LA measure in the evaluation of LV filling (Nagueh *et al*., 2016). Recent data on consecutive outpatients referred for echocardiography suggest that LAEF and LAVImin are stronger predictors of future cardiovascular events than LAVImax (Caselli *et al*., 2010). In addition, we recently showed that LAVImin was more strongly related to elevated NT‐proBNP than both LAVImax and the E/e′ ratio in a population‐based cohort (Hedberg *et al*., 2016). The present data demonstrated that LAVImin was more strongly related to LAEF than LAVImax, confirming a previous report from Abhayaratna *et al*. (2008) showing that a decline in LAEF was mainly driven by an augment in LAVImin. In contrary to LAVImax, LAVImin is directly exposed to LVEDP during atrial contraction and, therefore, LAVImin might be a more sensitive marker of an early augment in LVEDP than LAVImax. Overall this might suggest that if LA volume measures are used for either prognostic or diagnostic purpose in heterogeneous study populations, LAVImin or LAEF is to be preferred before LAVImax.

### Limitations

The study design, presenting cross‐sectional differences rather than longitudinal changes between serial measurements in individual patients, is a major limitation. Another major limitation is that invasive pressure measurements were not available, nor were pulmonary artery pressure or pulmonary venous flow. Our data are limited to adult Caucasian subjects with an AMI. A considerable number of patients were excluded mainly because of missing Simpson‐LVEF and LAV measurements. The excluded patients were more often women and had a larger body mass index compared with the included patients, which could be explained by the well‐known effect of breast tissue and overweight on echocardiographic image quality. Although the excluded patients did not differ from the included patients regarding age, smoking, hypertension, diabetes or previous CV disease, there could have been unmeasured differences which might be a cause of bias. The acquisition and storage of loops dedicated to LA planimetry were only obtained in the four‐chamber view, and not in the two‐chamber view. Thus, the guideline‐recommended biplane assessment of LA volumes was not possible, which is a limitation.

## Conclusions

A reduced LAEF was strongly associated with a lower reservoir volume index but higher LAVImax, LAVIpreA, LAVImin and LA transit volume indices. LAVImin was more strongly related to LAEF than LAVImax suggesting that a reduced LAEF was mainly driven by an augment in LAVImin. The TD‐a′ was strongly and linearly related to LAEF, whereas the MV‐A and the E/A ratio showed an association with LAEF only in lower levels of LAEF. The observed associations of LA volumes and Doppler filling indices with a reduced LAEF are likely a reflection of the adaptation to sustain the LV filling volume and counteracting a rise in pulmonary venous pressure in face of an enhanced LV end‐diastolic pressure.

## Conflict of interests

The authors have no conflicts of interest.
